# Birth Defects and Long-Term Neurodevelopmental Abnormalities in Infants Born During the Zika Virus Epidemic in the Dominican Republic

**DOI:** 10.5334/aogh.3095

**Published:** 2021-01-05

**Authors:** Raquel Pimentel, Shaveta Khosla, Josefina Rondon, Farah Peña, Gwyneth Sullivan, Martha Perez, Supriya D. Mehta, Maximo O. Brito

**Affiliations:** 1 Epidemiology Directorate, Ministry of Health, Santo Domingo, Dominican Republic; 2 Department of Emergency Medicine, University of Illinois at Chicago, Chicago, IL; 3 Department of Pediatric Neurology, Robert Reid Cabral Hospital, Santo Domingo, Dominican Republic; 4 Department of Surgery, Rush University, Chicago, IL; 5 Division of Epidemiology & Biostatistics, School of Public Health, University of Illinois at Chicago, Chicago, IL; 6 Division of Infectious Diseases, Department of Medicine, University of Illinois at Chicago, Chicago, IL

## Abstract

**Background:**

When acquired during pregnancy, Zika virus (ZIKV) infection can cause substantial fetal morbidity, however, little is known about the long-term neurodevelopmental abnormalities of infants with congenital ZIKV exposure without microcephaly at birth.

**Methods:**

We conducted a cross sectional study to characterize infants born with microcephaly, and a retrospective cohort study of infants who appeared well at birth, but had possible congenital ZIKV exposure. We analyzed data from the Dominican Ministry of Health’s (MoH) National System of Epidemiological Surveillance. Neurodevelopmental abnormalities were assessed by pediatric neurologists over an 18-month period using Denver Developmental Screening Test II.

**Results:**

Of 800 known live births from 1,364 women with suspected or confirmed ZIKV infection during pregnancy, 87 (11%) infants had confirmed microcephaly. Mean head circumference (HC) at birth was 28.1 cm (SD ± 2.1 cm) and 41% had a HC on the zero percentile for gestational age. Of 42 infants with possible congenital ZIKV exposure followed longitudinally, 52% had neurodevelopmental abnormalities, including two cases of postnatal onset microcephaly, during follow-up. Most abnormalities resolved, though two infants (4%) had neurodevelopmental abnormalities that were likely associated with ZIKV infection and persisted through 15–18 months.

**Conclusions:**

In the DR epidemic, 11% of infants born to women reported to the MoH with suspected or confirmed ZIKV during pregnancy had microcephaly. Some 4% of ZKV-exposed infants developed postnatal neurocognitive abnormalities. Monitoring of the cohort through late childhood and adolescence is needed.

## INTRODUCTION

Infection with ZIKV causes a relatively mild clinical disease in adults. When acquired during pregnancy, however, the disease can cause obstetric and neonatal complications [1]. The association between ZIKV infection and fetal neurological disease was observed during an outbreak in Brazil and in a retrospective evaluation of the French Polynesian outbreak [2,3]. Subsequent studies have confirmed a causal pathway for central nervous system damage and infection [4]. Pregnancy complications are, in most cases, unrelated to women’s symptomatology and appear to be most severe when infection occurs in the first trimester [1,5].

Microcephaly is a consequence of direct neurotoxicity and ZIKV invasion of the developing fetal brain [6]. Studies of early brain development, using the human brain organoid model, suggest that the virus preferentially infects human embryonic neural progenitor cells (NPC) [7]. The infection of NPCs, perhaps the most numerous cells in the developing embryonic/fetal central nervous system, leads to impaired neurogenesis, transcriptional dysregulation, attenuated cell growth, and apoptosis [8,9]. ZIKV activates the Toll-like-receptor 3-mediated immune response leading to genetic dysregulation and triggering apoptosis [10]. Further, ZIKV may cause brain growth failure from impaired neuro-angiogenesis resulting in delayed vascular development [11].

A relatively small proportion of infants born to women with ZIKV infection during pregnancy have birth defects (~6%) or neurodevelopmental problems (9%) [12,13]. Infants born without obvious morphologic abnormalities may develop other ZIKV-associated birth defects or long term neurodevelopmental abnormalities during the first 12–18 months of life.

Fetal and infant illnesses fall within a spectrum ranging from early fetal loss and stillbirths to the signature constellation of clinical signs in live births: the Congenital Zika Syndrome (CZS) [14,15]. Among the most salient features and radiographic characteristics of CZS are ocular abnormalities, such as macular scarring and retinal mottling, subcortical cerebral calcifications, contractures, hypertonia, and microcephaly [16,17]. Post-mortem neuropathological findings are well documented [18,19].

The introduction of ZIKV to the Dominican Republic (DR) occurred in early 2016. As we previously reported, the infection spread widely throughout the country with more than 5,000 symptomatic cases reported to the Ministry of Health (MoH) [20]. Among these cases, there were 1,282 pregnant women with suspected ZIKV infection. Among 788 women with recorded outcomes, 718 (91%) of those pregnancies were live births, 24 (3%) were stillbirths, and there were 46 (6%) miscarriages. There were only three confirmed cases of fetal microcephaly among these initial deliveries [21]. However, there were more than 200 infants born with suspected microcephaly reported to the MoH after the initial wave of ZIKV infections in the general population. These infants were born to asymptomatic or mildly symptomatic women who were not reported to the MoH while pregnant. In addition, there was a substantial number of infants born to women with laboratory evidence of ZIKV with possible congenital ZIKV exposure who appeared well at birth.

The objectives of this study were to describe the clinical and epidemiological characteristics of infants with Zika-associated microcephaly, and to report on the neurodevelopmental abnormalities during the first 18 months of life for a group of infants with possible congenital ZIKV exposure.

## METHODS

### STUDY DESIGN AND SETTING

The MoH instituted epidemiologic surveillance for ZIKV shortly after the onset of the epidemic with mandatory reporting of pregnant women with suspected or confirmed ZIKV infection, and infants born with congenital microcephaly. To support this, the MoH introduced a single reporting form to be completed by all public and private health centers countrywide. These reports were transmitted to the MoH via the National System of Epidemiological Surveillance (SINAVE), the online platform for individual case and outbreak reporting of the MoH. The first part of this study is a cross sectional analysis of data exported from SINAVE, *pregnant women with suspected Zika infection* and *infants born with congenital microcephaly*, to identify all cases of microcephaly reported during and after the countrywide outbreak of 2016–2017.

The second part of the study is a retrospective cohort analysis of infants exposed to ZIKV in utero who were followed at the neurodevelopmental clinics of the Robert Reid Cabral Children’s Hospital in the capital city of Santo Domingo and the Arturo Grullón Hospital in the city of Santiago during 2016–2019. This group of infants were born to women with confirmed ZIKV infection who had been reported to the MoH via the *pregnant women with suspected Zika infection* SINAVE database. Because our aim was to understand the potential post-natal neurodevelopmental abnormalities resulting from in utero ZIKV exposure, we only included in the retrospective cohort analysis infants born without evident Zika associated birth defects. Infants were followed at the neurodevelopmental clinics at 1, 2, 3, 6, 9, 12, 15, and 18 months. Expert pediatric neurologists performed comprehensive neurologic exams and conducted neurodevelopmental evaluations using the Denver Developmental Screening Test II (DDST), which evaluates four domains of child development: 1) personal social; 2) fine motor and adaptive; 3) language; and 4) gross motor [22,23].

#### Case Definitions

*Suspected ZIKV cases* were individuals with acute onset of rash and/or fever (>38.2ºC) and at least one of the following: arthralgia or myalgia; non-purulent conjunctivitis or conjunctival hyperemia; and headaches not explained by other medical conditions. *Probable ZIKV cases* were suspected cases with positive ZIKV IgM antibodies and no evidence of other arboviral diseases. *Confirmed ZIKV cases* were suspected cases with ZIKV RNA detected in urine or blood [24].

We used published surveillance case classification for children, neonate to 2 years, born to mothers with any evidence of ZIKV infection during pregnancy [13], to characterize: 1) Zika associated birth defects (i.e. microcephaly) and 2) exam findings of neurodevelopmental abnormalities possibly associated with congenital ZIKV infection such as: a) “hearing abnormalities”; b) “body tone abnormalities – hypotonia or hypertonia – in conjunction with a failed assessment for gross motor function”; c) “possible developmental delay (i.e. failed screen for gross motor domain or failed screen for ≥ 2 developmental domains at same time point or age)”; d) “possible visual impairment”; and e) postnatal-onset microcephaly (during two most recent head circumference [HC] measurements from follow up care using the World Health Organization [WHO] child growth standards). This surveillance case classification and others define *microcephaly* as head circumference (HC) < 3^rd^ percentile, adjusted for gestational age and sex, 24–48 hours after birth [25]. HC percentiles were determined using the standard growth chart of the International Fetal and Newborn Growth Consortium for the 21^st^ Century (INTERGROWTH-21^st^) [26,27]. We also used the Intergrowth 21^st^ Newborn size application tool to estimate percentiles and z-scores for HC by gestational age and sex. Pediatricians used WHO international growth charts to follow longitudinal growth parameters during post-natal visits [28].

#### Laboratory Testing

ZIKV IgM was obtained in children with microcephaly within 24–72 hours after birth. Dengue and Chikungunya testing was also performed to rule out co-infection or test cross reactivity. All mothers of the 42 children cohort tested positive for ZIKV reverse transcription-polymerase chain reaction (RT-PCR) while pregnant.

#### Data Source and Collection

For the cross-sectional analysis, we extracted de-identified surveillance data from SINAVE. The standardized microcephaly case report form included information on: infant sex, place of residence, gestational age at birth, care setting, mother signs/symptoms during pregnancy, mother ZIKV testing results, and HC measurement. For inferential analysis, we defined *severe microcephaly* as a HC at the zero percentile, adjusted for gestational age and sex.

For the retrospective cohort study of mother/infant dyads, data were abstracted from outpatient records of infants followed at the neurodevelopmental clinics. Region of residence was dichotomized as Greater Santo Domingo (capital city and Monte Plata Province) vs. all other provinces. Insurance status was categorized as having “any insurance” vs. “no insurance.” Birth was either premature (<37 weeks) or full term (≥37 weeks). Gestational age was estimated by date of last menstrual period.

### STATISTICAL ANALYSIS

Data from SINAVE and from the clinic visit database were imported to SAS 9.3 (SAS Institute, Cary, NC, USA) for analyses. We calculated frequencies for categorical variables, and measures of central tendency for continuous variables. We generated an epidemic curve of the outbreak by epidemiological week. We compared distributions of demographic and clinical findings by severity of microcephaly and used Chi-square, two sample t-test, and Wilcoxon rank sum test to obtain p-values for test of significance. We conducted log binomial regression to identify factors associated with severe microcephaly. Five cases were excluded from the multivariable analysis because their HC percentile at birth could not be confirmed. We geo- mapped cases and created the map on ArcGIS version 10.4.1 (ESRI, Redlands, CA, USA). For the retrospective cohort, we summarized neurologic abnormalities for each infant over time through a sequence plot created with Stata 13 (Stata Corp. 2013. College Station, TX). Proportional Venn diagrams representing neurodevelopmental and DDST abnormalities were generated using *eulerr* conducted in R statistical computing environment.

## ETHICS STATEMENT

The Institutional Review Board of the University of Illinois at Chicago and the National Council of Bioethics of the Dominican Republic approved the study protocol. Parents provided signed informed consent authorizing infant participation and follow up in the neurodevelopmental clinics.

## RESULTS

### ZIKV-ASSOCIATED MICROCEPHALY

**Figure 1:**
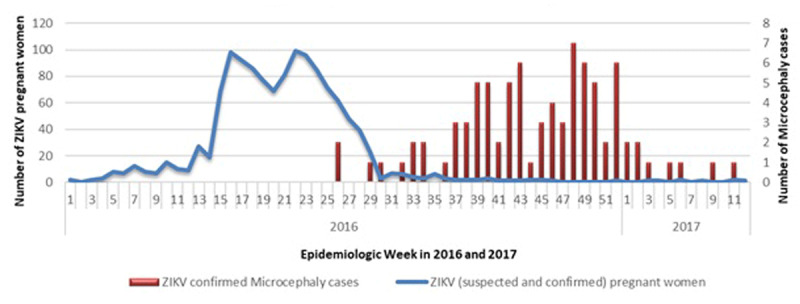
Epidemic curve of ZIKV cases among pregnant women and confirmed microcephaly cases in Dominican Republic, 2016–2017.

**Figure 2:**
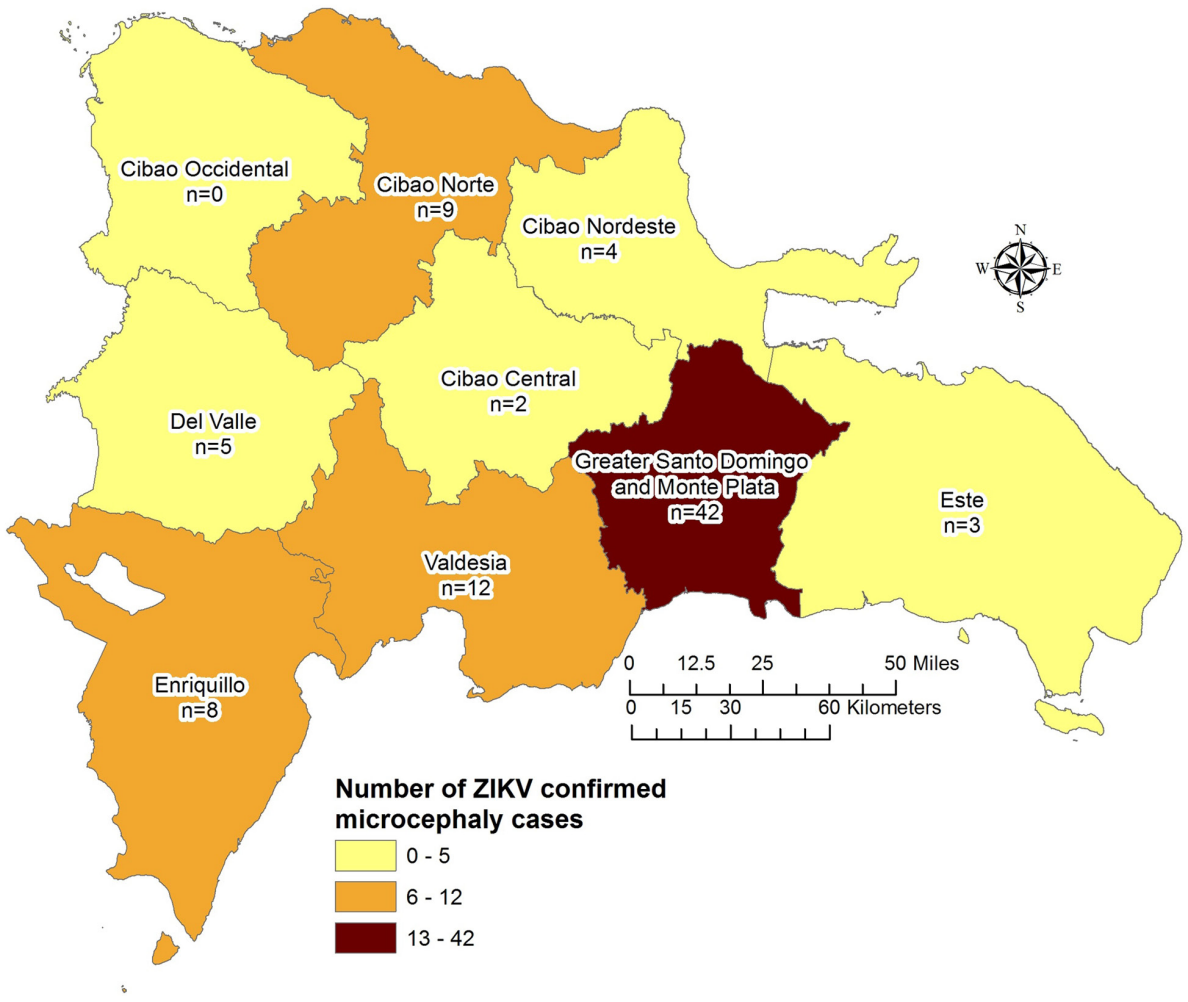
Cases of microcephaly by region, Dominican Republic 2016–2017.

We analyzed 800 live births resulting from 1,364 pregnancies, for which data on birth outcome were available, that were reported to the MoH via SINAVE. The MoH was unable to locate the remainder women to confirm pregnancy birth outcomes. Of these, 1,282 women were reported while experiencing ZIKV symptoms during pregnancy and 82 women and their infants were reported after delivery when the infant was noted to have microcephaly. Although there were 251 cases of probable microcephaly independently reported to the MoH through SINAVE between 2016 and 2017, only 184 met the case definition of microcephaly and the MoH was able to verify 85 of these cases. The remaining mothers could not be reached for confirmation of the infant’s HC measurement and/or to perform ZIKV testing of the infant, and thus, were not included in this analysis. Only three of the 85 infants with microcephaly were born to women who had been reported as suspected ZIKV infection during pregnancy. The rest were reported after birth as their mothers were asymptomatic or mildly symptomatic and were not captured by the surveillance system during pregnancy. The peak in ZIKV cases in the general population, and consequently in pregnant women, occurred between epidemiologic weeks 15 through 23 of 2016. Cases of microcephaly peaked at weeks 43 to 52 of the same year (Figure 1). The Greater Santo Domingo area had the highest number of cases (Figure 2).

More than half the infants (55%) were male (Table 1). The majority (89%) required inpatient care after birth and some (13%) had respiratory distress. One infant was born with a meningocele and one died soon after birth. The mean gestational age at birth was 37.8 weeks (± 1.95 weeks) and 13% were born prematurely, which is higher than DR’s estimated pre-term birth rate of 8 per 100 live births [29]. The mean HC was 28.1 cm (SD ± 2.1 cm). Severe microcephaly was detected in 67 (84%) cases. A substantial number (41%) had a HC on the zero percentile for gestational age.

**Table 1:** Characteristics of newborns with microcephaly.


	INFANTS WITH MICROCEPHALY N = 85 N (%)

**Sex**	

Male	47 (55.3)

Female	38 (44.7)

**Mother’s insurance status during pregnancy**	

Any	24 (31.6)

None	52 (68.4)

Unknown/Missing	9

**Country of origin**	

Dominican Republic	80 (94.1)

Haiti	4 (4.7)

Other	1 (1.2)

**Care setting**	

Ambulatory	3 (3.5)

Hospitalized	76 (89.4)

Unknown/Missing	6

**Complications**	

None	42 (54.6)

Difficulty breathing	10 (13.0)

Unknown	25 (32.5)

Missing	8

**Vital Status**	

Dead	1 (1.2)

Alive	84 (98.8)

**Gestational age**	

<32 weeks	1 (1.2)

≥32–36 weeks	10 (11.8)

≥37 weeks	74 (87.0)

**Mother’s Zika Status**	

Positive	40 (47.1)

Negative	6 (7.1)

Unknown	39 (45.8)


#### Maternal symptoms and microcephaly

Only 45 women (53%) recalled having ZIKV-like symptoms during pregnancy. Among symptomatic women, the most common symptoms were rash (n = 30, 67%), fever (n = 19, 42%), and arthritis/arthralgia (n = 11, 24%). ZIKV infection was confirmed by RT-PCR in 40 mothers, 39 had either an unknown or undetermined ZIKV status, and 6 mothers were ZIKV RT-PCR negative. Among 41 women who recalled the gestational age at symptom onset, 35 (85%) were symptomatic in the first trimester, 5 (13%) early in the second trimester, and 1 (2%) reported symptoms before becoming aware she was pregnant. One symptomatic and two asymptomatic women reported that their spouses had also experienced ZIKV-like symptoms during the epidemic.

#### Predictors of severe microcephaly

We examined demographic and pregnancy characteristics to identify potential predictors of severe microcephaly. As reported in Table 2, none of the analyzed variables were associated with severe microcephaly.

**Table 2:** Factors associated with severe microcephaly*.


	HC = 0 PERCENTILE N = 33	HC > 0 PERCENTILE N = 47	CRUDE PRR (95% CI)

**Sex**			

Male	20 (46.5%)	23 (53.5%)	1.32 (0.77–2.28)

Female	13 (35.1%)	24 (64.9%)	ref

**Region of residence**			

Greater Santo Domingo^¶^	14 (35.9%)	25 (64.1%)	0.77 (0.45–1.32)

All other provinces	19 (46.3%)	22 (53.7%)	ref

**Insurance**			

Any	7 (31.8%)	15 (68.2%)	0.71 (0.36–1.41)

None	22 (44.9%)	27 (55.1%)	ref

**Women confirmed ZIKV**+			

Yes	16 (43.2%)	21 (56.8%)	1.09 (0.65–1.84)

No/unknown	17 (39.5%)	26 (60.5%)	ref

**Preterm birth**			

Yes (32–36 weeks)	4 (40.0%)	6 (60.0%)	0.96 (0.43–2.17)

No (≥37 weeks)	29 (41.4%)	41 (58.6%)	Ref


* Five cases were excluded because head circumference measurement could not be determined/confirmed. ^¶^ Includes residents of Monte Plata Province. PRR = Prevalence Rate Ratio; CI = Confidence Interval; Ref = reference category.

### NEURODEVELOPMENTAL ABNORMALITIES POSSIBLY RELATED TO ZIKV INFECTION

We evaluated 42 infant/mother dyads followed at two pediatric neurodevelopmental clinics for 18 months. All mothers were symptomatic during pregnancy, had laboratory confirmed ZIKV infection by urine or serum RT-PCR, and their infants were born without obvious Zika-associated birth defects. Nearly half (n = 19; 45%) of the women were from the province of Santo Domingo and more than one fourth (n = 11; 26%) were from Santiago, the second largest province in the country. Table 3 describes the sociodemographic characteristics of the infants’ mothers.

**Table 3:** Sociodemographic characteristics of women with ZIKV during pregnancy who delivered infants without obvious ZIKV-associated birth defects (N = 42).


VARIABLES	MEAN ± SD OR N (%)

**Age**	

Mean ± SD (Range)	26.8 ± 4.9 (17–35)

**Age at first pregnancy**	

Mean ± SD	20.1 ± 3.7

**Number of pregnancies**	

Mean ± SD	2.9 ± 1.5

**Number of children**	

Mean ± SD	2.3 ± 1.2

**Previous child with congenital infection**	

Yes	2 (4.8)

No	40 (95.2)

**Family history of birth defects**	

Yes	3 (7.1)

No	39 (92.9)

**Sexually active during pregnancy**	

Yes	39 (92.9)

No	3 (7.1)

**Male partner experienced ZIKV- like symptoms**	

Yes	13 (31.0)

No	29 (69.0)

**History of smoking**	

Yes	5 (11.9)

No	37 (88.1)

**Current alcohol use**	

Yes	15 (35.7)

No	27 (64.3)

**Employed**	

Yes	20 (47.6)

No	22 (52.4)

**Work outdoors**	

Yes	19 (45.2)

No	23 (54.8)

**Timing of Zika symptoms**	

First trimester	21 (52.5)

Second or third trimester	19 (47.5)

Missing	2


**Figure 3:**
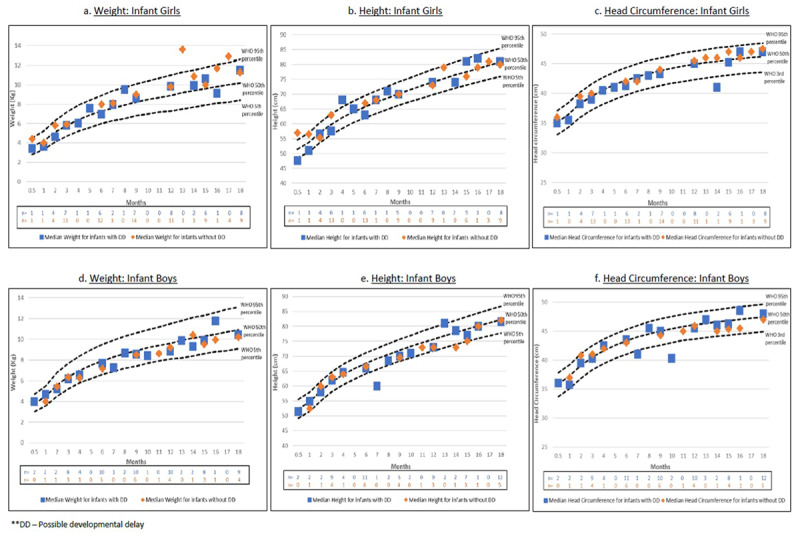
The distribution of weight, height, and head circumference among infant girls and boys.

More than half (n = 23; 55%) of the infants were girls. Figure 3 illustrates the estimated median of the most common growth parameters by gender, presence/absence of possible developmental delay, and clinic visit. Most infants, irrespective of the presence or absence of *possible developmental delay*, were around the 50th percentile of WHO’s charts for weight, height, and HC.

#### Postnatal-onset microcephaly

Two infants, one boy and one girl, developed post-natal microcephaly. The boy was diagnosed at the 1-month visit and had a CT of the brain which showed frontal calcifications, ventriculomegaly, and encephalomalacia. HC at the 1-month visit was 34.5 cm (<2^nd^ percentile for age and sex). As expected, he had abnormal DDST in all four domains throughout the observation period. His mother was 18 years old, had ZIKV infection during the first trimester of pregnancy, and this infant was her first born. She did not drink alcohol or use drugs, and did not experience any other pregnancy related complications. The girl was diagnosed at the 3-month visit and developed moderate-severe hypertonia and visual impairment. HC was 33 cm at 3 months of age. Her mother was 26 years old, had ZIKV infection during the first trimester of pregnancy, reported a past smoking history, occasional alcohol consumption, and no history of drug use. This infant was her third child. A third, female infant had a small HC for age, but did not meet criteria for microcephaly (HC between 25^th^–50^th^ percentile for age and sex). She developed hypertonia towards the end of the observation period.

#### Body tone abnormalities

**Figure 4:**
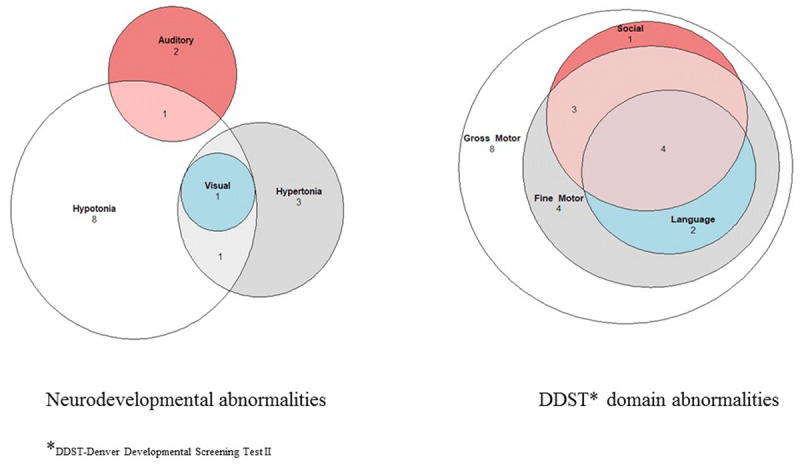
Proportional Venn Diagram of neurodevelopmental and DDST domain abnormalities.

The most common *body tone abnormalities* were hypotonia (n = 11) and hypertonia (n = 5). Most of these abnormal motor findings were detected in early infancy (n = 12 at 0–3 months and n = 6 at 3–6 months of life). Figure 4 illustrates how these body tone abnormalities overlap with other neurodevelopmental problems within the cohort. Two cases of hypertonia were detected during early visits and resolved with physical therapy. One of these cases was observed in an infant with normal DDST screening, and thus, the case did not meet the surveillance case classification criteria for a ZIKV-associated neurodevelopmental abnormality. Hypertonia was observed in the two infants with microcephaly described above. Most cases of hypotonia were mild, detected during the early visits, and improved over time, with 82% resolving by the last observed visit.

#### Hearing abnormalities

There were three infants with probable *hearing abnormalities*. One exhibited hypotonia and failed the language and social domains of the DDST at early visits. These abnormalities improved with therapy, but he continued to have problems in the language domain of DDST through the 15-month visit. Another infant failed the DDST social domain at some visits.

#### Possible visual impairment

There was one infant reported with visual impairment, who also exhibited hypertonia and hypotonia at different follow up visits.

#### Possible developmental delay

**Figure 5:**
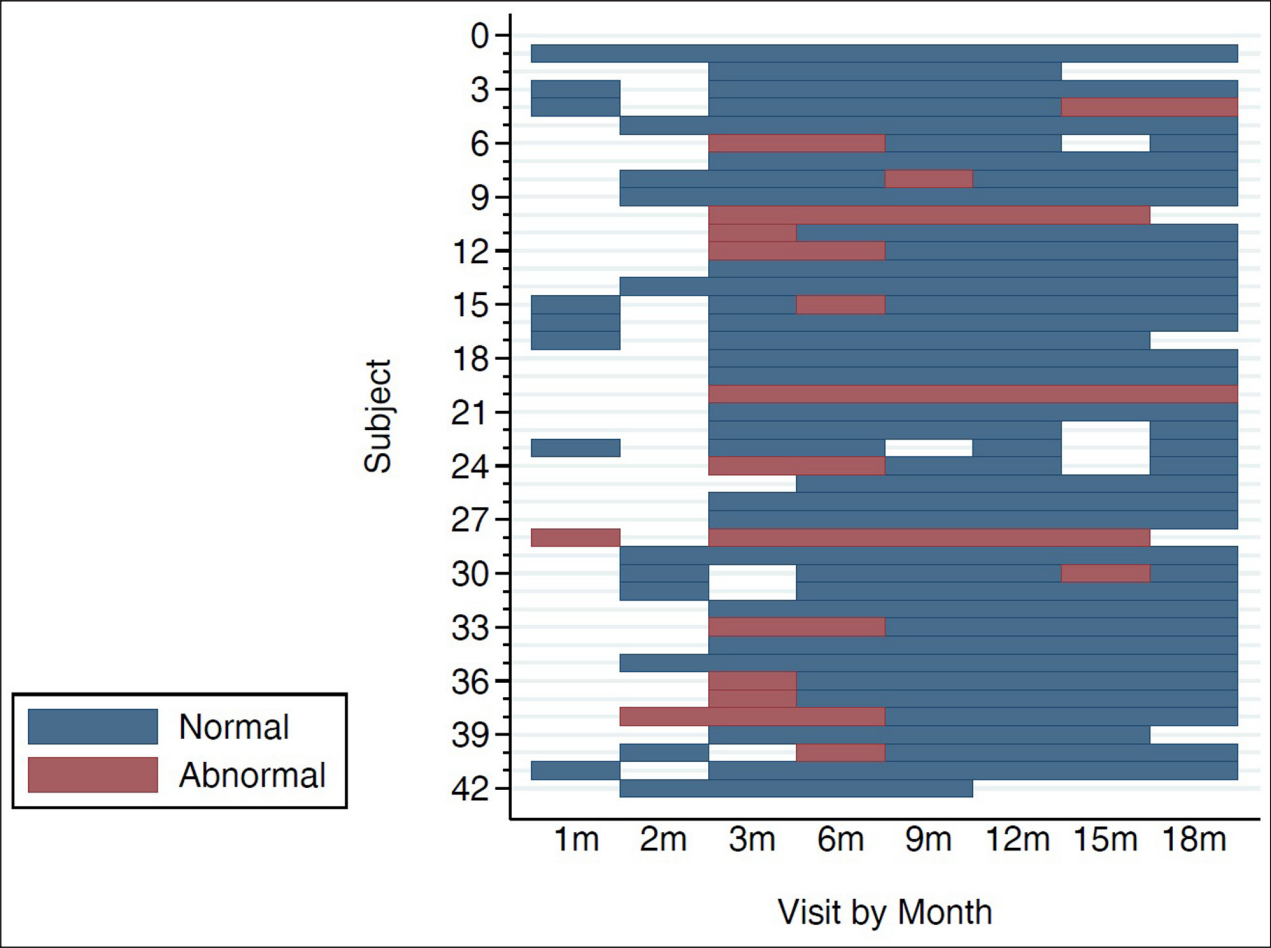
Sequence plot showing neurodevelopmental abnormalities by clinic visit.

Twenty-two (52%) of infants exhibited *possible developmental delay* as evidenced by an abnormal DDST screen in the gross motor domain at one or more visits. Of these, eight failed screening on the gross motor domain while the rest failed a combination of gross motor with fine motor, social and language domains (Figure 4). In 59% (13/22) of these cases the infant had an associated body tone abnormality, as described above, which was probably responsible for the failed DDST screen. For the majority of these infants, the developmental delay was mild and transient and most showed improvement in the milestones within each domain over time. Only four infants exhibited persistent neurodevelopmental abnormalities through the end of the observation period (Figure 5).

To evaluate the role of maternal characteristics on infant’s developmental delay we compared sociodemographic characteristics and clinical presentation of mothers with or without an infant with abnormal developmental screening and found no significant differences between the two groups except for a higher frequency abdominal pain during pregnancy in women whose infants had an abnormal developmental screen (85% vs. 38%, p = 0.007). Although the sample size is small, maternal alcohol use and smoking history were not associated with infant’s developmental delay.

## DISCUSSION

This report describes a cohort of 87 verified cases of infant microcephaly (85 congenital and 2 post-natal) and examines the longitudinal follow up of 42 infants with possible in-utero Zika exposure but no discernable associated birth defect. Most cases were reported from the capital city of Santo Domingo and the province of Monte Plata, mirroring the distribution of ZIKV among pregnant women reported previously [21]. These regions have the highest population densities in the country and reported the largest number of ZIKV infections in the general population. The larger concentration of people makes them more susceptible to explosive epidemics such as the ZIKV outbreak.

Since microcephaly may be independently associated with maternal and environmental factors other than ZIKV infection, we sought to identify potential factors that could produce a more severe disease. However, we did not find an association between the variables in the dataset and severe microcephaly. Because there were only a few variables available for this analysis, any conclusions about the degree of microcephaly severity and ZIKV infection are limited and need further study.

Of 1,364 pregnancies reported to the MoH as suspected or confirmed ZIKV infection, we were able to analyze data for 800 live births. Of these, 87 (11%) infants had microcephaly (85 congenital and 2 postnatal). This percentage is higher than expected due to the substantial number of pregnancies for which we do not know the outcome. Baseline prevalence of microcephaly in the DR, prior to the ZIKV outbreak, is unknown. Other countries in the Americas have retrospectively estimated baseline prevalence to better ascertain the impact of their respective epidemics. For instance, a meta-analysis conducted in Colombia estimated an average pre-ZIKV prevalence of microcephaly of 1.8 (95% CI 1.7–1.8) per 10,000 births [30]. Baseline risk in Puerto Rico, a neighboring Caribbean island, was estimated at 2–6 per 10,000 live births [31]. Since this analysis is based on limited passive surveillance data, it is not possible to estimate prevalence of ZIKV related microcephaly during the epidemic year.

The majority of microcephaly cases occurred 28–29 weeks after the epidemic peak in the general population, which included pregnant women (Figure 1), suggesting a temporal association with first trimester infections, which has been observed in other outbreaks [20,21]. The risk of microcephaly for first trimester infections was estimated at 1% in a previous ZIKV outbreak [32], but some reports suggest that risk could be as high as 13% [33]. In a case-control study conducted in Brazil, the matched odds ratio for women with first trimester ZIKV microcephaly was 73.1 [34]. The association between ZIKV and microcephaly remained strong after controlling for larvicide exposure and vaccine administration, which were alternative explanations for microcephaly posited early in the epidemic. In Colombia, prevalence of microcephaly increased significantly after the ZIKV outbreak to 9.6 per 10,000 births in 2016 [35].

Only half the mothers of infants with microcephaly recalled having ZIKV-like symptoms during pregnancy. The absence of symptoms should be interpreted with caution, however, since women were interviewed several weeks or months after the potential exposure, which increases the possibility of poor recall. Nonetheless, we know that CZS can occur in asymptomatic maternal infections. The ratio of asymptomatic to symptomatic infections varies by study. An earlier prevalence study estimated the ratio at 4.4:1; however, subsequent longitudinal cohort studies suggest the ratio may be closer to 1:1 [12,36,37]. The most common symptoms reported by women in this cohort – rash, fever, and arthralgia – are consistent with other published studies [38].

Of 42 infants with possible congenital ZIKV exposure followed longitudinally, 52% exhibited possible developmental delay in at least one visit throughout the 18-month observation period. Mothers of infants with developmental delay were more likely to experience abdominal pain during pregnancy, which may have been a sign of an unrecognized obstetric complication. However, we are unable to confirm this hypothesis and validate this observation using these retrospective data. Interestingly, most of the observed neurodevelopmental abnormalities resolved over time and only four infants were noted to have abnormalities that persisted for 15–18 months. In our opinion, these infants had ZIKV-associated neurodevelopmental abnormalities. It is possible that some of the remaining 18 infants that exhibited developmental delay at some point during the observation period had a true subtle ZIKV-associated neurodevelopmental problem; however, we could not evaluate that hypothesis in this analysis. Therefore, it is necessary to continue to follow these infants beyond childhood and through adolescence to detected subtle developmental problems that could arise with the passage of time. If we exclude the two infants with microcephaly, there were 5% (2/42) of infants with neurodevelopmental abnormalities possibly associated with congenital ZIKV infection, which is slightly lower than the 9% reported in the literature [13]. Our cohort may approach that 9% if some of the infants with transient neurodevelopmental abnormalities end up developing more permanent findings as they grow up. Therefore, we do not think our numbers differ significantly from what has been observed in other cohorts and ZIKV epidemiological outbreak analyses. Another regional cohort of Colombian infants exposed to ZIKV in utero without obvious physical abnormalities at birth also found infants with social cognition and mobility abnormalities during the first 18 months of life [39]. More than 30% of the infants developed post-natal brain imaging abnormalities, which the authors posit could have played a role in the pathogenesis of the developmental delay [40]. Of 404 infants born to mothers with confirmed ZIKV infection and monitored for the first 12 months of life in New York, United States, seventeen (4.2%) had a possible ZIKV-associated neurodevelopmental abnormality. Of these, seven were diagnosed with CZS and ten were noted to have a neurodevelopmental abnormality shortly after birth or at follow up [41].

Some of the limitations of the cross-sectional analysis are inherent to working with data extracted from passive surveillance systems. First, there is little information on the mother’s health during pregnancy given that most cases were not reported during pregnancy. Second, women were retrospectively questioned about ZIKV related symptoms introducing the possibility of recall bias and recall limitation. Third, the absence of microcephaly prevalence data prior to the epidemic precludes an accurate estimation of the epidemic’s impact at the population level. Although some could argue that the higher concentration of cases in the capital city may represent reporting bias, a condition of such dramatic presentation as microcephaly is unlikely to be unreported regardless of the province where it occurred. The limitations of our retrospective cohort analysis are the lack of brain imaging which limits our ability to fully characterize the impact of ZIKV exposure on infants’ central nervous system and a small sample size, which limits our power to assess meaningful differences.

In conclusion, there was an increase in cases of microcephaly reported via passive surveillance during the ZIKV epidemic in the DR and we observed that 4% of a small infant cohort born to Zika infected mothers had neurodevelopmental abnormalities during the first 18 months of life. The epidemic exposed weaknesses in the surveillance of pediatric neurological diseases prompting the standardization of head circumference measurement protocols and the institution of active surveillance countrywide. It is imperative to continue the longitudinal follow up and surveillance of infants born to women with Zika during pregnancy to detect subtle neurocognitive problems in late childhood and early adolescence.
